# Measuring underreporting and under-ascertainment in infectious disease datasets: a comparison of methods

**DOI:** 10.1186/1471-2458-14-147

**Published:** 2014-02-11

**Authors:** Cheryl L Gibbons, Marie-Josée J Mangen, Dietrich Plass, Arie H Havelaar, Russell John Brooke, Piotr Kramarz, Karen L Peterson, Anke L Stuurman, Alessandro Cassini, Eric M Fèvre, Mirjam EE Kretzschmar

**Affiliations:** 1 Centre for Immunity, Infection and Evolution, Ashworth Laboratories, Kings Buildings, University of Edinburgh, Edinburgh, UK; 2 Julius Centre for Health Sciences and Primary Care, University Medical Centre Utrecht, Utrecht, the Netherlands; 3 Department of Public Health Medicine, School of Public Health, University of Bielefeld, Bielefeld, Germany; 4 Centre for Infectious Disease Control, National Institute for Public Health and the Environment, Bilthoven, the Netherlands; 5 Institute for Risk Assessment Sciences, Utrecht University, Utrecht, the Netherlands; 6 European Centre for Disease Prevention and Control, Stockholm, Sweden; 7 Pallas, Health Research and Consultancy BV, Rotterdam, the Netherlands; 8 International Livestock Research Institute, Nairobi, Kenya; 9 Institute of Infection and Global Health, University of Liverpool, Liverpool, UK

**Keywords:** Underestimation, Underreporting, Under-ascertainment, Surveillance, Infectious diseases

## Abstract

**Background:**

Efficient and reliable surveillance and notification systems are vital for monitoring public health and disease outbreaks. However, most surveillance and notification systems are affected by a degree of underestimation (UE) and therefore uncertainty surrounds the 'true’ incidence of disease affecting morbidity and mortality rates. Surveillance systems fail to capture cases at two distinct levels of the surveillance pyramid: from the community since not all cases seek healthcare (under-ascertainment), and at the healthcare-level, representing a failure to adequately report symptomatic cases that *have* sought medical advice (underreporting). There are several methods to estimate the extent of under-ascertainment and underreporting.

**Methods:**

Within the context of the ECDC-funded Burden of Communicable Diseases in Europe (BCoDE)-project, an extensive literature review was conducted to identify studies that estimate ascertainment or reporting rates for salmonellosis and campylobacteriosis in European Union Member States (MS) plus European Free Trade Area (EFTA) countries Iceland, Norway and Switzerland and four other OECD countries (USA, Canada, Australia and Japan). Multiplication factors (MFs), a measure of the magnitude of underestimation, were taken directly from the literature or derived (where the proportion of underestimated, under-ascertained, or underreported cases was known) and compared for the two pathogens.

**Results:**

MFs varied between and within diseases and countries, representing a need to carefully select the most appropriate MFs and methods for calculating them. The most appropriate MFs are often disease-, country-, age-, and sex-specific.

**Conclusions:**

When routine data are used to make decisions on resource allocation or to estimate epidemiological parameters in populations, it becomes important to understand when, where and to what extent these data represent the true picture of disease, and in some instances (such as priority setting) it is necessary to adjust for underestimation. MFs can be used to adjust notification and surveillance data to provide more realistic estimates of incidence.

## Background

Efficient and reliable surveillance and notification systems are vital for monitoring public health trends and disease outbreaks. They also often form the backbone of evidence-based decision-making processes, as well as infectious disease (ID) public health policies that deal with prioritisation, and the planning of intervention measures and healthcare services [1]. However, there are limitations associated with the use of data from surveillance and notification systems since most systems are affected by a degree of underestimation and therefore uncertainty surrounds the 'true’ incidence of disease [2]. IDs are considered particularly prone to underestimation due to their specific characteristics (e.g. asymptomatic or self-limiting disease courses) and are therefore represented inadequately by raw surveillance data. Thus, when routine data are used to inform decisions relating to resource allocation or to estimate epidemiological parameters in a population, it becomes important to understand when, where and to what extent these data do or do not comprehensively represent the true picture of disease. Furthermore, in certain circumstances, such as priority setting, it is appropriate to adjust infectious disease datasets in order to account for the portion not captured by the surveillance system. There are several metrics that can be employed in priority setting with Disability-Adjusted Life Years (DALYs) being just one composite health metric that combines and measures adverse health effects and premature mortality in a single unit. DALYs were chosen by the European Centre for Disease Prevention and Control (ECDC) and used within the Burden of Communicable Diseases in Europe (BCoDE)-project to generate evidence-based and comparable burden of disease (BoD) estimates for 32 IDs across European Member States (MS) [3-6]. A major prerequisite of DALY calculations is 'true’ incidence data but since data are often obtained from (inter)national-level routine surveillance datasets that are frequently incomplete, data must be adjusted before serving as input for computing disease burden.

**Figure 1:**
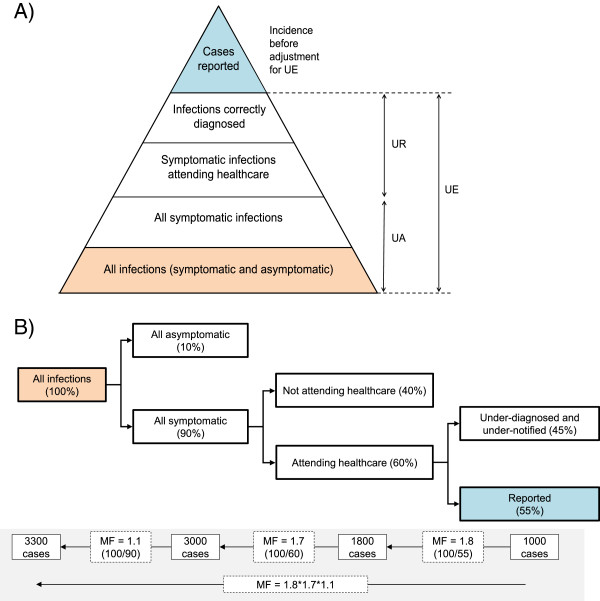
**Deriving multiplication factors from the morbidity surveillance pyramid. A**: The morbidity surveillance pyramid is often used to illustrate the availability of morbidity data at each surveillance level. With each ascending level (from the community, to healthcare institutions (GPs, hospital, laboratory), to regional and national public health agencies); data availability shrinks and only a fraction of cases from the level below is captured [7-9]. In contrast to the narrow tip of the pyramid which represents data held by national public health agencies, the base is wide as it holds all infections in the community. The difference between the number at the tip and base can be considered cases lost to 'underestimation’ (UE). **B**: The proportions of infections that are symptomatic, that attend healthcare, and that are reported are represented in this decision tree model. Here, only 55% of all infected individuals attending healthcare are reported through the notification system. If 1000 cases were reported then a MF of 1.8 (=100/55) could be derived and would correct for those underreported cases. The true number attending healthcare would be 1800 cases. Likewise, if only 60% of symptomatic cases attended healthcare, then a MF of 1.7 (=100/60) would correct for under-ascertainment of symptomatic cases. The true number of cases attending healthcare would be 3000 symptomatic cases (=1.7*1800). Finally, since 90% of infections were symptomatic, a MF of 1.1 (=100/90) would correct for under-ascertainment of asymptomatic cases. The true number of infections would be 3300 (=1.1*3000). A MF to correct for total underestimation of symptomatic cases in one step would be 3.06 (=1.8*1.7) and for all infections 3.4 (=1.8*1.7*1.1). 'All infections’ shaded in orange in Figure 1A represents the same population as the orange box in Figure 1B. 'Cases reported’ in blue in Figure 1A represents the same population as the blue box in Figure 1B.

Here we present an overview of why, where and in what form underestimation occurs within the morbidity surveillance pyramid (Figure 1A) and we give several disease-specific examples from the literature of the methods that can be used to estimate the extent of underestimation. Furthermore, we compare the extent of underestimation and multiplication factors to adjust for it using key examples from the literature for two diseases, salmonellosis and campylobacteriosis. This body of work was a core aspect of the BCoDE-project.

### Definitions

Underestimation (UE), as defined here, can be understood as the many ways in which surveillance systems fail or are unable to reflect all infections in a given population. Mathematically, UE is the number of infections estimated to have occurred in a population that have not been captured by the surveillance system for every reported case over a given time period. UE can be split into two distinct levels as represented by the surveillance pyramid for IDs (Figure 1A); under-ascertainment (UA) of infections occurring at the community-level and underreporting (UR) of infections occurring at the healthcare-level. Under-ascertained infections occur in individuals that do not seek healthcare and hence cannot be captured by surveillance systems which are typically designed to capture cases that do seek healthcare. UA can be estimated as the number of infections occurring in individuals that do not attend healthcare services for every case that attends. There is a symptomatic fraction of all under-ascertained cases that do not attend healthcare due to mild symptoms and/or the knowledge that the illness is self-limiting or for some other reason, and an asymptomatic fraction that do not seek healthcare as they are not aware of their infection status due to lack of symptoms [10]. Underreported infections are infections in individuals that do seek healthcare, but whose health event is not captured by the surveillance system and not notified through the notification system [7,8,11,12]. UR can be estimated as the number of infected individuals attending healthcare services whose health event is not reported to the appropriate public health body for every attending case whose health event is reported. UR can be due to under-diagnosis which accounts for the cases attending healthcare but whose infection or pathogen is not diagnosed or misdiagnosed [7,8], and under-notification which accounts for the failure to report (using correct International Classification of Diseases (ICD) codes [13,14]) all positive diagnoses through the notification system [15,16]. Reporting completeness refers to the proportion of cases attending healthcare whose health event was correctly diagnosed and appropriately reported [17]. These technical terms are used frequently in the literature, however often with varying definitions. The definitions of UA, UR and UE as stated here were developed during the BCoDE-project [3,18] and will be used as such for the remainder of the article.

### Factors influencing UA in morbidity datasets

Not all people who are infected with a pathogen seek healthcare [19]. One important reason for this is that when symptoms are absent, mild or self-limiting, there is a lack of urgency to seek healthcare [8]. Therefore, surveillance systems can only capture cases with symptoms that are severe enough to motivate infected individuals to attend healthcare services. Health literacy also influences the decision to attend healthcare services or not. If a community has a poor understanding of when to seek healthcare and lacks knowledge of the severity or duration of an illness; then the uptake of healthcare services and levels of case ascertainment could be lower than expected. Such awareness and recognition of disease as well as the urgency or perceived need to seek healthcare can vary through time and space, particularly during outbreak years and especially if there is enhanced surveillance or widespread campaigns and intensive media coverage [20]. Compared with non-outbreak years, the proportion of cases in a population that is ascertained is expected to be greater. Health literacy and perceived need for healthcare may also explain often observed differences between age- and sex-specific ascertainment rates with, for example, children aged less than 15 years being statistically more likely to seek healthcare for gastroenteritis compared to adults (30–64 years) [21].

In addition, cultural and religious factors could prevent individuals from seeking healthcare if, for example, there is stigma or negative beliefs associated with healthcare services, illness and treatment [22,23]. There may also be legal, administrative and financial barriers to attending healthcare if individuals are not registered or are unable to register and if healthcare is dependent on the legal status of an individual or ability to pay. Migrants or marginalised groups may be particularly affected by this [24] and in addition to not having their disease episode captured by the surveillance system, they may not be enumerated at all and hence do not contribute to the country population count or denominator. Individuals from remote communities and their illness may also be uncounted due to healthcare services being physically unreachable. Overall, ascertainment rates are thought to vary significantly within and between disease groups, population groups and countries.

### Factors influencing UR in morbidity datasets

Not all cases that attend healthcare will have their health status correctly diagnosed and reported to the appropriate health authorities. This break in the surveillance chain can occur within clinics, hospitals or laboratories due to healthcare workers lacking in ability, capacity or knowledge of how and when to act. Under-diagnosis may arise when biological samples are not requested from or provided by patients, where there are budget restrictions forcing healthcare professionals to limit their requests for testing samples, lack of knowledge of which tests to perform, inadequate diagnostic tools, or due to restrictions of laboratory testing regimes (regulations on which tests to apply routinely, and lack of availability of more specialised tests). Under-notification may result from an inadequate reporting system or lack of knowledge of when, for which diseases and how to report correctly including knowledge of ICD codes [25-29]. The proportion of cases reported is often higher where there is a legal requirement to report (some diseases or pathogens have mandatory reporting statuses) [30] or where there are incentives for healthcare workers to request or test biological samples from patients or to report results [26]. Furthermore, where there is a perceived urgency to request biological samples or report cases, for example during outbreak years where there is higher awareness and chance of recognition and motivation for testing [31,32], reporting rates are likely to increase. In contrast, UR may be greater for rarer diseases, those with only occasional outbreaks or those without mandatory reporting statuses. This perceived urgency or necessity to test or report tends to increase for more serious conditions (severity and duration of illness [8,33]) and can also be age- or sex-dependent [34]. Incomplete reporting of additional information, such as age and sex of the patient, concurrent infections or sequelae following an initial infection [35] becomes particularly important when UR for a particular disease is age- or sex-specific.

### Identifying areas and extent of UE

Various study designs can be used to determine the extent of UR and UA in the surveillance system.

#### Community-based studies

Community-based studies (CBS) aim to generate new estimates of pathogen carriage or infection in a (representative) sample of the population. This alone is useful and interesting but in addition, if this new estimate is considered the 'true’ incidence in the community, it can be compared to notification data and the magnitude of UE deduced. This order of magnitude can then be used as a multiplication factor (MF) (Figure 1B) to adjust disease datasets assuming that the base value to which the MF is being applied was created using the same data type (i.e. MF calculated by comparing CBS data with notification data is used to adjust other notification datasets, and not laboratory data). These observational studies can also be used to produce incidence rates of symptomatic and asymptomatic cases as well as estimate the specific proportion of symptomatic cases presenting to healthcare facilities (by asking about attendance in the questionnaire). Furthermore, the proportion of cases underreported can be estimated by a variation of CBS in which healthcare professionals are surveyed to gain information on propensity to request biological samples and reporting habits.

CBS can take many forms but generally involve active searching within the community for disease episodes, pathogen carriage or infection, with questionnaire-based data acquisition often accompanied by biological sampling. Active searching can be conducted face-to-face, by telephone, internet or post, with several possible study designs e.g. based on probability samples, prospective or retrospective cohorts, population cross-sections, involving representative samples of the whole population or certain interest or high-risk groups only. CBS are especially useful for diseases commonly under-ascertained (i.e. those with many mild and asymptomatic cases of mostly self-limiting illness) and where an unknown burden exists within the community, e.g. sexually transmitted infections with *Chlamydia trachomatis*[36-40] or *Neisseria gonorrhoea*[36-39,41-44]; influenza and influenza-like illnesses [45,46]; and food and water-borne diseases [2,47-54]). The first and second Infectious Intestinal Disease studies (IID1 and IID2) were prospective community cohort studies that estimated overall incidence of infectious intestinal disease (IID) in the UK community, the proportion seeking healthcare (ascertained), and the proportion reported to the national public health agency [2,11,47,55]. Weekly surveys (by email or prepaid postcard) recorded if participants had experienced diarrhoea and/or vomiting and if they had, they were asked additional questions and requested to provide a stool sample. Similarly, several studies have employed statistical and mathematical methods to estimate incidence and UE using data collected during a Dutch prospective community-based cohort study of gastroenteritis (SENSOR) and a Dutch GP-based cohort of gastroenteritis (NIVEL) (e.g. [56-63]).

CBS are not without limitations as bias can arise at numerous points. Sampling bias, owing to non-random sampling of a population, can result in a study that is not representative of the entire population with certain groups (such as ethnic, migrant, age or occupational) inadvertently excluded from the study because they are unregistered, not easily locatable, do not have access to a telephone (in the case of telephone surveys), have language barriers or are marginalised for other reasons. Responder bias can also lead to unrepresentative samples since only certain groups of people will agree to participate, and measurement bias can result from case definitions that are undefined, too general, too strict or simply not used consistently. Additionally, interviewers may ask questions or interpret responses in a leading manner or respondents may induce bias during disease occurrence recall. To minimise the effects of bias, Wilking *et al.*[54] took several steps in a recent population-based telephone survey of acute gastrointestinal illness in Germany including; contacting listed and unlisted telephone numbers and using the 'last-birthday method’ to reduce responder bias, using the computer-assisted telephone interview (CATI) method to minimise interviewer bias and applying study weights to improve representation of the target population. Telephone surveys, however, have a further limitation associated with them since disease occurrence tends to be based on self-reported symptoms and so without clinically-determined or laboratory-confirmed diagnoses, there is uncertainty surrounding the causative agent and incidence of infection. To address this, Kubota *et al.*[50] used data from population-based telephone surveys to adjust the number of cases for each pathogen from active laboratory-based surveillance (whilst modelling for uncertainty). Other telephone surveys focus on general conditions, such as gastroenteritis, rather than specific pathogens and these have been successfully applied in many countries (e.g. [54,64-66]) with the results often the basis for pyramid reconstruction activities (see below).

#### Serological surveys

Serological surveys are a specific type of CBS that measure sero-incidence (the rate of new infections) or sero-prevalence (the total number of infections in the community or cohort) as quantified by antigen or antibody positivity. This CBS can capture asymptomatic and symptomatic, historical and acute infections but if biological sampling is combined with a questionnaire asking about disease episodes, or if the antibody or antigen threshold at which symptoms manifest is known; then the symptomatic fraction can be obtained (e.g. hepatitis B [67], hepatitis B and C [68], pertussis [69], measles [70], HIV [71]). This is crucial for BoD studies since, for the majority of diseases, it is only clinical manifestations and not asymptomatic infections that contribute to burden in terms of DALYs. The exceptions to this are the few infectious diseases with a possible asymptomatic acute stage that can result in sequelae (and death) at a later time and hence contribute to disease burden (including hepatitis B, hepatitis C, chlamydia, Invasive Meningococcal Disease, and Q-fever). When calculating DALYs for these diseases, symptomatic cases serve as input to estimate the numbers of asymptomatic infections and thus the calculated burden attributable to asymptomatic infections is included in the final burden estimate [4].

It is often difficult to differentiate between historic and current infections and therefore it is important to test for recognised serological markers of recent infection and to have full knowledge of antibody decay rates in different populations [72,73]. Furthermore, antibodies resulting from natural exposure versus vaccination cannot be distinguished and thus serological surveys of diseases with universal vaccine coverage, including tuberculosis (BCG vaccine), measles, rubella, and other childhood vaccine-preventable diseases, may have limited use.

#### Returning traveller studies (RTS)

Returning traveller studies (RTS) are further examples of CBS where individuals returning from abroad represent sentinel populations for the reported national incidence of infection in a traveller’s destination of travel [74]. In RTS, the risk of infection for travellers from country A visiting country B is calculated by taking the number of infected travellers returning home from country B from surveillance records as a numerator, and the total number of travellers from country A visiting country B from travel pattern databases as the denominator. This measure of risk can then be used to generate a new estimate of incidence in country B (risk multiplied by the population size), which when compared to the national notification records of country B, can generate a MF of underestimation. Using this method, the incidence and proportion of underestimated cases of salmonellosis in several European countries was calculated by de Jong and Ekdahl [75] by comparing the incidence of infection in Swedish returning travellers to the national incidence in the countries the travellers had returned from, using Norway as a reference country. This not only produced national-level comparable estimates of incidence, but also a MF of UE (or in this case 'under-detection index’). More recently, Havelaar *et al.*[76] calculated incidence rates of salmonellosis and campylobacteriosis and MFs based on disease risks of returning Swedish travellers, anchored to data from SENSOR, the Dutch population-based study on gastroenteritis (see Tables 1 and 2).

**Table 1:** A comparison of multiplication factors (MFs) for salmonellosis in several countries

**Country**	**Underestimation MF**	**Primary study type**	**Study**	**Under-ascertainment MF**	**Primary study type**	**Study**	**Underreporting MF**	**Primary study type**	**Study**
Austria	3	RTS	[75]						
Austria	11	RTS	[76]						
Belgium	1.9	RTS	[75]						
Belgium	3.5	RTS	[76]						
Bulgaria	271	RTS	[75]						
Bulgaria	718.5	RTS	[76]						
Croatia	30.6	RTS	[75]						
Cyprus	71.2	RTS	[75]						
Cyprus	173.2	RTS	[76]						
Czech Republic	3	RTS	[75]						
Czech Republic	28.9	RTS	[76]						
Denmark	1.8	RTS	[75]						
Denmark	4.4	RTS	[76]						
Denmark	17	PRC	[9]						
Denmark	NT UEinf: 325 (5-95% quartiles: 190–505)	Sero/MOD	[77]						
Estonia	1.3	RTS	[75]						
Estonia	16.9	RTS	[76]						
Finland	0.6	RTS	[75]						
Finland	0.4	RTS	[76]						
France	8.3	RTS	[75]				*S.entd.* $ : Data1: 7.1(95% CI: 6.7-7.7)	CRS	[78]
							Data2: 12.5 (95% CI: 11.1-14.3)		
							Data3: 2 (95% CI: 1.9-2.2)		
							*Spp* other than *S.entd.& S.typh.*		
							Data1: 12.5 (95% CI: 7.1-16.7)		
							Data2: 16.6 (95% CI: 10–25)		
							Data3: 2.0 (95% CI: 1.1-2.8)		
France	26.9	RTS	[76]						
Germany	1.8	RTS	[75]						
Germany	9.8	RTS	[76]						
Germany	6.7	PRC	[9]						
Greece	97.7	RTS	[75]				1.75	MOD/RC	[79]
Greece	1228.5	RTS	[76]						
Greece	51.45 (PERT: 3.2; 99.7)	BoD/CRS	[80]						
Hungary	5.5	RTS	[75]						
Hungary	66.8	RTS	[76]						
Ireland	4.3	RTS	[75]						
Ireland	5.4	RTS	[76]						
Italy	13.1	RTS	[75]						
Italy	71.7	RTS	[76]						
Italy	17	PRC	[9]						
Latvia	11.7	RTS	[75]						
Latvia	44.3	RTS	[76]						
Lithuania	10	RTS	[75]						
Lithuania	59.1	RTS	[76]						
Luxembourg	4.5	RTS	[76]						
Malta	92.6	RTS	[75]						
Malta	222.7	RTS	[76]						
Poland	16.2	RTS	[75]						
Poland	114.1	RTS	[76]						
Poland	18	PRC	[9]						
Portugal	378	RTS	[75]						
Portugal	2082.9	RTS	[76]						
Romania	332	RTS	[75]						
Romania	349.9	RTS	[76]						
Slovakia	3.5	RTS	[75]						
Slovakia	53.2	RTS	[76]						
Slovenia	10	RTS	[75]						
Slovenia	40.3	RTS	[76]						
Spain	103	RTS	[75]						
Spain	214.2	RTS	[76]				NT: Data 1 = 2.0 (95% CI: 2.0 - 2.1)	CRS	[81]
							Data 2 = 1.5 (95% CI: 1.4 - 1.5)		
Sweden	0.5	RTS	[76]						
Sweden	10	PRC	[9]				Data 1 =1.05, Data 2 =1.02	CRS	[82]
The Netherlands	7.7	RTS	[75]						
The Netherlands	26.3 (ref)	RTS	[76]						
The Netherlands	24.7 (5-95% quartiles: 5.2 - 64.7)	BRI/BoD	[57]	5.8 (5-95% quartiles: 0.8 - 25.6)	BRI	[57]	4.3 (5-95% quartiles: 2.5 - 6.5)	BRI	[57]
The Netherlands	14 (5-95% quartiles: 3.6 – 56)	CBS/BoD	[56]	6.5 (5-95% quartiles: 0.0 - 20)	CBS/BoD	[56]			
The Netherlands	14.3	LAB	[62]						
The Netherlands	20	PRC	[9]						
United Kingdom	4.3	RTS	[75]						
United Kingdom	7.3	RTS	[76]						
United Kingdom	4.7 (95% CI : 1.2 - 18.2)	CBS	[47]	3.4 (95% CI: 0.4 - 32.2)	CBS	[47]	1.4 (95% CI: 0.6 - 3.3)	CBS	[47]
United Kingdom	3.2 (95% CI : 1.4 - 12.0)	CBS	[2]	GP only, 1.4 (95% CI: 0.7 - 2.8)	CBS	[2]			
United Kingdom	40	PRC	[9]						
United Kingdom	NT, UElab: 3.9	CBS/BoD	[55]						
EU-27 (excl.Croatia)	57.5 (11–140)	RTS	[76]						
Iceland	0.4	RTS	[75]						
Norway	1.0 (ref)	RTS	[75]						
Norway	1.2	RTS	[76]						
Switzerland	7.1	RTS	[76]						
USA	NT, UElab: BD 9.8, NBD 67.7, total 38.6	CBS/BoD	[49]	NT, BD 6.8, NBD 8.6	[49]				
USA	NT, 38 (taken from [49])	BoD	[83]	NT, BD - 2.86 (PERT 1.96 – 5.26)	BoD	[84]	NT UN, 1		[84]
				NBD – 5.56 (PERT 5–6.67)					
Canada	13 - 37	PM	[85]						
Australia	‡BD:1-2d: 11.39 (95% CrI: 8.49–16.36)	PM	[33]	‡BD: 1-2d: 10 (95% CrI: 7.1-14.3)		[33]			
	3-4d: 2.82 (95% CrI: 2.17–3.98)			3-4d: 2.3 (95% CrI: 1.9-3.2)					
	≥5d: 1.81 (95% CrI: 1.33–2.72)			≥5d: 1.5 (95% CrI: 1.1-2.2)					
	NBD: 1-2d 143.29 (95% CrI: 83.3–371)			NBD; 1-2d: 10 (95% CrI: 7.1-14.3)					
	3-4d 13.06 (95% CrI: 6.37–67.83)			3-4d: 2.3 (95% CrI: 1.9-3.2)					
	≥5d 3.93 (95% CrI: 2.10–11.92)			≥5d: 1.5 (95% CrI: 1.1-2.2)					
	Overall: 7 (95% CrI: 4–16)								
Japan	74.0 (5-95% quartiles: 35.8, 140.7)	CBS	[50]	*S.brae.* Age <10 years: 1.2,	CBS	[48]			
				> = 10 years: 1.7, Overall:1.6,					

1. This table lists all extracted or derived MFs (with variance shown as 95% CI, 95% CrI, PERT distribution (max, min, mode), or 5-95% quartiles where available) from relevant studies found during the extensive literature review. MFs give an estimation of the extent of UE (combined UA and UR), UA and UR for salmonellosis in a particular country; the higher the MF, the higher the proportion of cases not captured by the surveillance system. These MFs could be applied to official figures as reported by public health agencies to adjust for UE and give a new estimate of total symptomatic infections occurring in a population at a given time. Exceptions include; “UEinf” where the MF can adjust official figures from public health agencies and give a new estimate of total infections (both symptomatic and asymptomatic) occurring in a population at a given time, and “UElab” where the MF can adjust official laboratory figures of laboratory confirmed infections and give a new estimate of total infections (both symptomatic and asymptomatic) occurring in a population at a given time. MFs of UA and UR can be multiplied together to make one MF of UE.

2. Study types abbreviations: CBS: Community-based study, RTS: Returning traveller study, CRS: Capture-recapture study, PRC: Pyramid reconstruction model, BRI: Bayesian risk of infection model, BoD: Burden of disease calculation, Sero: Analysis of serology data, LAB: Analysis of laboratory surveillance, RC: Analysis of reporting completeness, PM: Probability model, OUT: Outbreak analysis, MOD: Modelling other. Symptoms abbreviations: NT: Non-typhoidal salmonellosis; NBD: Non-bloody diarrhoea; BD: Bloody diarrhoea; severity of diarrhoea (d = days). *Salmonella* species abbreviations: *S.entd.* : *S. enteritidis; S.typh. : S.typhimurium; S.brae. : S.braenderup.*Other abbreviations: UN: Under-notification of laboratory confirmed infection; GP only: cases attending GP surgeries (not hospitals) only; $ Estimates corrected by the positive predictive value of one data source where (unlike the other two sources) notifications are not validated by a systematic procedure; ‡ No. cases in the community for every 100 reported.

3. For CRS, a MF is given to correct for UR for each data source (i.e. MF for 'Data 1’ will estimate the underreporting in data source 1). For MFs estimated in the same RTS, one country will be listed as the reference country (i.e. 'ref’) and all other countries compared to this.

**Table 2:** A comparison of multiplication factors (MFs) for campylobacteriosis in several countries

**Country**	**Underestimation MF**	**Primary study type**	**Study**	**Under-ascertainment MF**	**Primary study type**	**Study**	**Underreporting MF**	**Primary study type**	**Study**
Austria	15	RTS	[74]						
Austria	29	RTS	[76]						
Belgium	25	RTS	[74]						
Belgium	11	RTS	[76]						
Bulgaria	39,000	RTS	[76]						
Cyprus	310	RTS	[76]						
Czech Republic	11	RTS	[76]						
Denmark	4	RTS	[74]						
Denmark	4.1	RTS	[76]						
Denmark	29	PRC	[9]						
Estonia	13	RTS	[76]						
Finland	1.0 (ref)	RTS	[74]	GP *C.jejuni* , 5.2	CBS/OUT	[86]			
Finland	0.4	RTS	[76]						
France	3,958	RTS	[74]						
France	280	RTS	[76]						
Germany	6	RTS	[74]						
Germany	4.4	RTS	[76]						
Germany	9.3	PRC	[9]						
Greece	47,191	RTS	[74]						
Hungary	52	RTS	[76]						
Ireland	46	RTS	[74]						
Ireland	29	RTS	[76]						
Italy	660	RTS	[76]						
Italy	100	PRC	[9]						
Lithuania	40	RTS	[76]						
Luxembourg	19	RTS	[74]						
Luxembourg	3.9	RTS	[76]						
Malta	90	RTS	[76]						
Poland	4,100	RTS	[76]						
Poland	72	PRC	[9]						
Romania	6,900	RTS	[76]						
Slovakia	35	RTS	[76]						
Slovenia	14	RTS	[76]						
Spain	270	RTS	[76]						
Sweden	0.4	RTS	[76]						
Sweden	17	PRC	[9]						
The Netherlands	31	RTS	[74]						
The Netherlands	22 (ref)	RTS	[76]						
The Netherlands	22.9 (5-95% quartiles: 8.2 - 50)	BRI/BoD	[57]	4.1 (5-95% quartiles: 9.3 – 56.7)	BRI/BoD	[57]	5.6	BRI/BoD	[57]
The Netherlands	10.9 - 21.4	Sero	[58]	5.0 - 5.4	Sero	[58]	2.0 - 4.3	Sero	[58]
The Netherlands	9.7 (5-95% quartiles: 4.1 - 23.0)	CBS / BoD	[56]	4.2 (5-95% quartiles: 0.0 - 7.4)	CBS/BoD	[56]			
The Netherlands	18.9	LAB	[62]						
The Netherlands	49	PRC	[9]						
The Netherlands	UElab, 13.94 (PERT 4.96 – 28.67)	MOD	[61]						
United Kingdom	11	RTS	[74]						
United Kingdom	4.4	RTS	[76]						
United Kingdom	9.3 (95% CI: 6–14.4)	CBS	[47]	7.2 (95% CI: 3.3 - 15.9)	CBS	[47]	1.3 (95% CI: 0.9 - 1.8)	CBS	[47]
United Kingdom	52	PRC	[9]						
United Kingdom	UElab 10.3	CBS /BoD	[55]						
United Kingdom	7.6 (95% CI: 3.6 - 17.4)	CBS	[2]	2.1 (95% CI: 5–3.0)	CBS	[2]			
EU-27	47 (range 0.4 - 39,000)	RTS	[76]						
Norway	4	RTS	[74]						
Norway	2.4	RTS	[76]						
Switzerland	3.3	RTS	[76]						
USA	38 (taken from [49])	BoD	[83]	NT, BD: 2.9 (PERT 2.0-5.3)	BoD	[84]	NT UN, 1	BoD	[84]
				NBD: 5.6 (PERT 5–6.7)					
USA									
Canada	23 - 49	PM	[85]						
Australia	‡BD: 1-2d 12.40 (95% CrI: 9.16– 17.82)	PM	[33]	‡BD: 1–2 days: 10 (95% CrI: 7.1-14.3)	PM	[33]			
				3-4 days: 2.3 (95% CrI: 1.9-3.2)					
	3-4d 3.06 (95% CrI: 2.32–4.33)								
				≥5 days: 1.5 (95% CrI: 1.1-2.2)					
	≥5d 1.97 (95% CrI: 1.42–2.95)			NBD: 1–2 days: 10 (95% CrI: 7.1-14.3) 3–4 days: 2.3 (95% CrI: 1.9-3.2)					
	NBD: 1-2d 154.2 (95% CrI: 89.3–397.6)								
				≥5 days: 1.5 (95% CrI: 1.1-2.2)					
	3-4d 14.15 (95% CrI: 6.80–73.32)								
	≥5d 4.25 (95% CrI: 2.25–13.36)								
	Overall: 10 (95% CrI: 6.6–22)								

1. This table lists all extracted or derived MFs (with variance shown as 95% CI, 95% CrI, PERT distribution (max, min, mode), or 5-95% quartiles where available) from relevant studies found during the extensive literature review. MFs give an estimation of the extent of UE (combined UA and UR), UA and UR for campylobacteriosis in a particular country; the higher the MF, the higher the proportion of cases not captured by the surveillance system. These MFs could be applied to official figures as reported by public health agencies to adjust for UE and give a new estimate of total symptomatic infections occurring in a population at a given time. Exception is; “UElab” where the MF can adjust official laboratory figures of laboratory confirmed infections and give a new estimate of total infections (both symptomatic and asymptomatic) occurring in a population at a given time. MFs of UA and UR can be multiplied together to make one MF of UE.

2. For abbreviations and symbols, see footnote 2 for Table 1.

3. For MFs estimated in the same RTS, one country will be listed as the reference country (i.e. 'ref’) and all other countries compared to this.

While RTS generate comparable estimates of incidence and multipliers across different countries, there are several assumptions and limitations. Even if the travel patterns database is representative of the whole population and that the origin of infection, as reported in surveillance data, is correct [74]; in calculating risk of infection, the numerator (number of infections caught abroad) will still be affected by UR and UA. This in part is due to general reasons affecting UE, but in addition travellers have different health-seeking behaviour than that of the general population and there may be bias in requesting samples and reporting by health professionals following travel to certain destinations [74]. Furthermore, in the case of Swedish travellers the duration and reason for travel differs by country, with many short business trips to neighbouring Scandinavian countries but longer holidays to Mediterranean countries [76] and therefore there is a bias in the risk estimates towards countries with the most tourists [75]. The risk of infection differs given the destination of travel but in addition the probability of the infection being ascertained also differs since on a longer trip, the case may have recovered before returning home and attending healthcare [76]. Lastly, a traveller’s risk is not the same as the native population’s risk due to differences in behaviours and activities, as well as immunity to local pathogen populations [76].

#### Capture-Recapture Studies (CRS)

**Figure 2:**
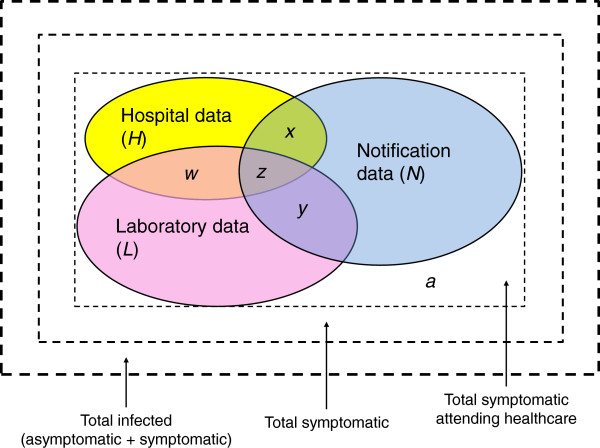
**Illustration of a three source capture-recapture study.** The outermost square represents the total number of infections occurring in a given population in a given time period, the second square represents the total symptomatic cases, and the innermost square represents all symptomatic cases attending healthcare. In this example, of all infected individuals attending healthcare, all cases - *a* will appear in at least one data source (which in this example are the laboratory database, hospital database and notifications sent to the public health agency through the notification system). *a* represents the number of symptomatic cases attending healthcare that were not captured by any data source and remain undiagnosed or not notified (i.e. the underreported cases). *x*, *y, w* and *z* cases are recorded in more than one data source with *x*, *y* and *w* captured in two data sources and *z* cases captured in 3 data sources. The true number of cases attending healthcare and that should be reported to the national level is: = cases in *N* + (cases in *H* (-*w* -*x* -*z*)) + (cases in *L* (-*w* -*y* -*z*)) + *a*. Adapted from: [87].

Capture-recapture studies (CRS) utilise the ecological principle for studying populations of wildlife by marking subjects on initial release or first capture and recovering information from them on subsequent captures [87-89]. In terms of human disease surveillance, a personal identifier number or code usually represents the 'marker’ and the 'captures’ are records of disease episodes, colonisations or infections found in data sources including national notifications of morbidity and death, hospital and GP records, laboratory reports, as well as other public health registries [87,90]. Two or more data sources are compared (Hest *et al.* stated that at least three are preferred to avoid correlations [90]) or cross-linked (through personal identifiers) and duplicates removed to approximate reporting completeness of each data source, identify the cases that would have been missed if using only a single data source and calculate a new estimate of incidence (Figure 2). There are several examples of CRS studies in the literature spanning a wide range of both communicable and non-communicable diseases (e.g. CRS of tuberculosis in several countries; Greece [79,91], Italy [92,93], the Netherlands [94,95], Romania [96], UK [97-99] and USA [17,100,101]). Despite the usefulness of this method, some cases attending healthcare are not captured in any single data source or are not correctly recorded and hence the new incidence of cases will still be affected by UR [89]. In addition, CRS only correct for UR of infections and do not account for UA occurring in the community.

#### Modelling

There are numerous mathematical and statistical methods that can generate new estimates of incidence in the population as well as calculating the predicted proportion of UE occurring at several steps of the reporting chain, which can then be used to generate country- and disease-specific MFs. As well as using simulated data, these methods often utilise data from national surveillance records, CBS, CRS and several other study designs and therefore it is difficult to consider modelling as an independent method for identifying UE (many studies use a combination of methods, e.g. statistical modelling is often used to analyse results of CBS).

Attack rates, that estimate the proportion infected (from which cumulative incidences can be estimated and MFs generated), are calculated using data from CBS or national surveillance data (although these are still subject to UE), (e.g. influenza [102,103]). Vaccine coverage, when below a certain threshold and when the basic reproduction number is known for a pathogen, can be used to estimate the number of susceptible individuals in the community and therefore expected incidence (and hence MFs), (e.g. measles [104]). Scallan *et al.*[105] and Thomas *et al.*[106] used statistical methods to 'scale-up’ counts of laboratory-confirmed cases to an estimated number of illnesses in the United States and Canada respectively, therefore adjusting for UE. Serological data can be modelled statistically to estimate incidence (e.g. HIV [107]) and past incidence of infection calculated from the current prevalence of antibody in a population using a catalytic model (e.g. hepatitis A [108]). Decision tree models, (e.g. for Sexually Transmitted Infections (STIs) [109], hepatitis A [110]), and similarly probability models and pyramid reconstruction models (e.g. food and water borne disease [9,33,58,85,111,112], influenza [19]) estimate the country- and pathogen-specific probabilities of action at each incremental stage of the surveillance pyramid (e.g. attending healthcare versus not, submitting a sample versus not, reporting versus non-reporting). Further modelling techniques include Bayesian synthesis of multiple evidence sources that estimate the 'true’ incidence of an infection at several steps of the surveillance pyramid, as well as changes in contact patterns and health-seeking behaviour (e.g. H1N1 influenza pandemic [113,114]). Simulation models, based on outcome trees of disease progression are also tools that can estimate expected incidence, (e.g. hepatitis B [115]).

## Methods

An extensive literature review was conducted to identify studies (of designs presented above) that estimate ascertainment or reporting rates for salmonellosis and campylobacteriosis in European Union Member States (MS), plus European Free Trade Area (EFTA) countries Iceland, Norway and Switzerland and all other OECD countries. Articles were considered relevant if they: measured the sensitivity of reporting or reported the rate of UA, UR or UE; reported MFs, measured a new incidence or prevalence of infection from which a MF could be derived; or used any alternative methodology to correct surveillance or notification data. To identify appropriate studies, a literature review for each disease (salmonellosis and campylobacteriosis) and each pathogen (*Salmonella* spp. and *Campylobacter* spp.) was conducted in PubMed using the search terms: burden, cost-of-illness, cost of disease, cost-effectiv*, cost-analys*, cost-benefit, cost-utility, disability-adjusted, mathematical model*, multiplication factor*, multiplier*, outbreak*, prospective stud*, quality of life, quality-adjusted, serological stud*, serological survey*, serosurveillance, sero-surveillance, seroprevalence, statistical model*, telephone (*denotes any ending to the search term); linked by 'OR’. The search was restricted to articles written in English and to the years 1990–2011 since surveillance systems, reporting protocols and epidemiological patterns may have been different in the years preceding 1990, hence MFs would be less appropriate for adjusting current surveillance and notification data.

Following identification of these studies, MFs were either taken directly from the literature or derived where the proportion of underestimated, under-ascertained, or underreported cases was known (MF = 100/(percentage reported or ascertained or estimated), Figure 1B). MFs for salmonellosis and campylobacteriosis were compared to gain an understanding of variation between and within countries when using different methods to estimate UR and UA.

## Results

MFs were found or derived for all European Union Member States (MS) plus European Free Trade Area (EFTA) countries Iceland, Norway and Switzerland and four other OECD countries (USA, Canada, Australia and Japan) for salmonellosis from 22 references, and similarly for campylobacteriosis (excluding Croatia, Greece, Iceland, Latvia and Portugal) from 18 references. Table 1 (salmonellosis) and Table 2 (campylobacteriosis) present MFs for adjusting surveillance data for UE in one step, and MFs for adjusting for UA and UR in these countries. By multiplying together one MF of UR and one MF of UA, this results in one single MF of UE. MFs were found to vary widely between study types, countries and diseases with MFs for UE ranging from 0.4 (suggesting over-reporting) [75] in Iceland to 2082.9 [76] in Portugal for salmonellosis and from 0.4 [76] in Sweden and Finland to 39,000 [76] in Bulgaria for campylobacteriosis. In countries with mandatory notification of infection (for salmonellosis this includes all EU countries other than Belgium, France, Luxembourg and Spain which are voluntary, and the UK which requires reporting of the pathogen rather than disease), reporting rates were expected to be higher (and hence MFs lower). Unfortunately, there are too few MFs of UR to verify this from these studies. The most common study type for generating MFs of UE for both diseases identified by the literature review was RTS which can provide comparable multipliers for several countries. Furthermore, most studies provide a single MF to adjust for UE in one step, suggesting this is the most straight-forward approach. Few studies (12 for salmonellosis and 10 for campylobacteriosis) provide any measure of uncertainty surrounding the MF. While age-stratified MFs are preferred since ascertainment and reporting are affected by age; stratification into age bands was only found for UA of salmonellosis (*S.braenderup*) in Japan in one study [48]. For individuals less than 10 years of age, the MF was lower than for individuals over 10 years and hence the older age group was ascertained less often. No study described sex-specific stratification of MFs for either disease. Two studies stratified MFs by severity of clinical symptoms based on duration of symptoms [33] and if there was blood in stool samples [33,49]. Where duration of illness was short, cases were less likely to seek healthcare leading to higher UA and higher MFs. With bloody diarrhoea due to *Salmonella* in USA, Voetsch *et al.*[49] estimated higher ascertainment (lower MF) and lower overall underestimation compared to non-bloody diarrhoea. Few studies make a distinction between strain types. Where MFs of UR were estimated from CRS (two studies of salmonellosis), a MF for each data source is listed showing the degree of UR per data source which is consistent for both studies. The United Kingdom and the Netherlands had the most number of MFs of UE for both salmonellosis and campylobacteriosis. For each country, all studies were reasonably consistent in terms of order of magnitude. This may be due to the overlap of data sources used in the studies. However, the highest MF estimates for both countries and each disease were estimated by the pyramid reconstruction model. These higher estimates of MFs may represent the thorough nature of accounting for each incremental step of the surveillance pyramid which could lead to double-counting of cases.

## Discussion

Here, we discuss the advantages and disadvantages of different methods for identifying UE in the surveillance pyramid and compare MFs resulting from those methods. MFs show considerable between-country and -disease variation which may reflect true differences in reporting and ascertainment rates. However, study design (which often depends on the disease, data type, data quality and availability of resources) and hence the method used to estimate UE likely accounts for the presented within-country variability of MFs. It remains difficult to select the most appropriate study (and corresponding MFs) as there are limitations associated with each. CBS that are representative of the whole population are often favoured for approximating UE, UA, and UR but the quality of MFs derived depends highly on the study design; CRS are very good at estimating UR in the surveillance pyramid but some reported cases may remain undetected and UA is not considered; and RTS and serological surveys also estimate UE effectively but the many associated limitations must be realised. In addition, an important limitation is the uncertainty that surrounds estimates which is relevant to all study types. Many of the studies from the literature review do not report uncertainty for MFs which gives a false impression that we are sure that these point estimates are correct. In fact, a great level of uncertainty is expected [33]. Since the factors contributing to UE are multiplicative (Figure 1B), they explode rapidly and the resulting MF can be very large (this is clearly observed in pyramid reconstruction studies which aim to account and correct for UE at each incremental step of the surveillance chain). Therefore, even small degrees of uncertainty in measuring individual components of UE can lead to wide ranges in incidence estimates and MFs. To estimate predictive intervals, uncertainty can be modelled by incorporating, for example, either uniform or pert probability distributions (rather than fixed point estimates), and using techniques such as Monte Carlo simulations [4,50,80,84].

One systematic way to decide on the best method for estimating UE or choosing MFs is to use the Delphi method or expert consensus. In the next phase of the BCoDE-project, internal ECDC experts with final input from the consortium and other external experts will create lists of the most appropriate country- and disease-specific MFs for 32 IDs. In general we assume that the most appropriate MFs should be disease-, country-, age-, and sex-specific because underestimation rates are disproportionately distributed between diseases, countries with differing surveillance systems and reporting procedures, and between demographic groups. While our literature review returned only one result with age- or sex-stratification of MFs, there are other studies that provide age- (at least for given age bands) [12,21,34,116-119] and sex-specific [117,118] MFs for general gastroenteritis and diarrhoea (i.e. unspecified pathogen). However, there remains a paucity of age- and sex-specific MFs in the literature.

Where no MF exists, it is (under certain conditions) possible to 'borrow’ or extrapolate from a disease of similar epidemiology or from the same disease in a country with a similar surveillance system, or likewise apply the same MF to a group of diseases (as demonstrated by Mead *et al.*[83]). However, it must be acknowledged that the base value of “cases reported” (Figure 1A and B) that we seek to adjust for UE by applying an appropriate MF, may not always capture the same proportion of infections that have occurred or provide comparable information of disease incidence estimates for different diseases or countries. Therefore, borrowing MFs (particularly from different countries and especially from different disease groups (e.g. taking MFs for STIs and applying to gastroenteric disease data)) is not a favoured method owing to the inherent heterogeneity of national surveillance systems in terms of population covered, test sensitivity and specificity, the source of data (physician, laboratory, hospital or other) and surveillance type (whether compulsory versus voluntary reporting of positive results or cases, comprehensive versus sentinel, active versus passive surveillance, case-based versus aggregated reporting [120]).

Here we did not address UE in the mortality reporting chain. Similar to the surveillance pyramid for morbidity data, the tip of mortality pyramid represents the cases correctly reported. The wide base of the pyramid contains data of all deaths including those that are ascertained and those that are not. Whilst it is expected that in a European setting under-ascertainment of deaths is rare (if not irrelevant), underreporting or over-reporting of mortality events due to certain diseases or conditions is not. The number of deaths may be well reported, but there is considerable misclassification of the cause of death. This misclassification may be deliberate in countries without nationalised healthcare such as the United States, where reimbursement by private insurance may be related to the ICD code used for the primary cause of death. Elsewhere, there may be other reasons to misclassify the cause of death, such as government targets and pressure to reduce the number of deaths due to a certain cause. In addition, often lacking are additional details relating to underlying conditions and sequelae that an individual died *with* (e.g. secondary and tertiary causes) but not necessarily *of* (i.e. the primary cause of death) [13,121,122]. For example, chronic conditions with infectious causes (e.g. liver cirrhosis) are often not counted as sequelae deaths and therefore the surveillance system may underestimate the long-term burden due to the infection that led to the sequelae.

## Conclusion

UE masks the true magnitude of disease incidence and reduces the efficiency of the notification system and surveillance potential [123]. In some instances, such as BoD estimates for the BCoDE-study and for comparing the impact of diseases between countries, it is necessary to quantify and adjust for UE. After correction for UE, preferably by age and sex, surveillance and notification data become a better estimate for evidence-based and comparable disease burden estimations. However, since adjusting for UE results in higher disease burden estimates and can result in diseases with differing ranks of public health importance compared with unadjusted surveillance data; care should be taken to clearly communicate both the need for such adjustment and the methodologies applied to adjust the raw data. The results presented here confirm that UR and UA have a significant impact resulting in UE of surveillance and notification data in our examples for salmonellosis and campylobacteriosis. To a varying extent, this is also true for all other pathogens in the BCoDE-study. The BCoDE-project is currently compiling and verifying estimates of UE and MFs derived from extensive literature reviews for 32 IDs. Here, we have presented several viable approaches for estimating UE and MFs of salmonellosis and campylobacteriosis although the best option will undoubtedly vary between countries.

## Competing interests

Alessandro Cassini and Piotr Kramarz (co-authors) are employed by the European Centre for Disease Prevention and Control, which has funded this research. The authors declare that they have no competing interest.

## Authors’ contributions

All authors contributed to the development of the methodology. CLG, M-JJM and ALS performed literature reviews and MF calculations. All authors read and approved the final version of the manuscript.

## Pre-publication history

The pre-publication history for this paper can be accessed here:

http://www.biomedcentral.com/1471-2458/14/147/prepub
